# Effects of salvianolic acid A on intestinal microbiota and lipid metabolism disorders in Zucker diabetic fatty rats

**DOI:** 10.1186/s13098-022-00868-z

**Published:** 2022-09-20

**Authors:** Xufeng Wang, Xiangjun Sun, Abulikemu Abulizi, Jinyao Xu, Yun He, Qian Chen, Ruicheng Yan

**Affiliations:** 1 Department of Gastrointestinal Surgery, Hubei Provincial Hospital of Traditional Chinese Medicine, 856 Luoyu Rd, Hongshan District, Wuhan, 430061 Hubei China; 2 Hubei Province Academy of Traditional Chinese Medicine, Wuhan, 430074 Hubei China; 3 Affiliated Hospital of Hubei University of Chinese Medicine, Wuhan, 430074 Hubei China

**Keywords:** Salvianolic acid A, Diabetes mellitus, Zucker diabetic fatty, Intestinal microbiota, Lipid metabolism, Insulin

## Abstract

Salvianolic acid A (SalA) is the main water-soluble component isolated from *Salvia miltiorrhiza*. This study explored the influences of SalA on intestinal microbiota composition and lipid metabolism in Zucker diabetic fatty (ZDF) rats. The 6-week-old male ZDF rats were treated with distilled water (N = 10) and low dose (SalA 0.5 mg/kg/d, N = 10), medium dose (SalA 1 mg/kg/d, N = 10), and high dose (SalA 2 mg/kg/d, N = 10) of SalA, with the male Zucker lean normoglycemic rats of the same week age as controls (given distilled water, N = 10). The blood glucose, body weight, and food intake of rats were examined. After 7 and 8 weeks of continuous administration, oral glucose tolerance test (OGTT) and insulin tolerance test (ITT) were performed, respectively. Serum fasting insulin (FINS), total cholesterol (TC), triglyceride (TG), and free fatty acid (FFA) were determined. Liver tissues were stained using hematoxylin–eosin (HE) and oil red O staining. Fecal samples were analyzed by 16S rRNA gene sequencing. Small intestinal tissues were stained using HE and immunohistochemistry. The tight junction proteins (ZO-1/Occludin/Claudin-1) and serum levels of LPS/TNF-α/IL-6 were evaluated. SalA reduced insulin resistance, liver injury, serum FFA, liver TC and TG levels in ZDF rats, and improved lipid metabolism. After SalA treatment, intestinal microbiota richness and diversity of ZDF rats were promoted. SalA retained the homeostasis of intestinal core microbiota. SalA reduced intestinal epithelial barrier damage, LPS, and inflammatory cytokines in ZDF rats. Overall, SalA can sustain intestinal microbiota balance and improve the lipid metabolism of ZDF rats.

## Introduction

Diabetes mellitus (DM) is a complicated metabolic disorder featured by hyperglycemia that results from defects in insulin secretion and insulin action and is related to alterations in protein, fat, and carbohydrate metabolism [1, 12]. According to epidemiological surveys, approximately 451 million people suffer from DM worldwide in 2017, and it is anticipated that 693 million DM cases occur globally by 2045 [6]. DM is generally classified into gestational DM, type 1 DM, and type 2 DM (T2DM), with T2DM covering approximately 90% of DM cases worldwide [2]. T2DM is mainly characterized by insulin resistance (IR) and impaired insulin secretion of pancreatic β cells, with the manifestations of obesity and disorders of glucose and lipid metabolism [38], indicating the tight association between IR and the onset and development of T2DM. Reducing blood glucose and improving IR are imperative for clinical T2DM treatment [56, 65]. To explore T2DM pathogenesis, male Zucker diabetic fatty (ZDF) rats are extensively used as they can aptly simulate the obesity-related T2DM occurrence and progression [53]. Hence, we selected ZDF rats as study subjects to investigate the mechanism of DM and provide a reference for treatment.

Salvianolic acid A (SalA) represents the major bioactive component in *Salvia miltiorrhiza* that is a traditional oriental medicine that is broadly applied for preventing and treating liver and cardiovascular diseases [25, 26, 62]. SalA possesses antioxidant [55], anti-inflammatory [14], and brain protection properties [58]. Furthermore, SalA has been unveiled to have anti-diabetic effects and can prevent diabetic complications [33, 54], revealing the promising therapeutic effect of SalA on DM.

Recently, alterations in intestinal microbiota composition and diversity are documented to be associated with DM progression [19]. For instance, the abundance of intestinal microbiota in diabetic rats is lower than that in normal rats [52]. The expression of Occludin, an intestinal barrier protein, is reduced in diabetic mice, indicating increased intestinal permeability [4]. T2DM patients exhibit prominently decreased intestinal microbiota richness and diversity and increased intestinal permeability and abundance of several opportunistic pathogens [3]. Traditional Chinese medicine can improve intestinal microbiota mainly by enhancing the richness and diversity of microbiota, increasing beneficial bacteria, and decreasing the relative abundance of harmful bacteria [29]. Moreover, preceding research reveals that SalA can effectively alleviate abnormal glucose and lipid metabolism, which exerts a paramount protective effect on diabetic peripheral neuropathy [54]. SalA improves intestinal motility in diabetic rats by anti-oxidation and upregulation of neuronal nitric oxide synthase activity [64]. However, the regulation of SalA in ZDF rats remains unclear. To explore the alleviating effect of SalA on hyperinsulinemia in ZDF rats, we hypothesized that SalA may play a role in maintaining the balance of intestinal microbiota and improving the pathway of lipid metabolism in ZDF rats. Therefore, we intended to investigate the action of SalA on intestinal microbiota and lipid metabolism in ZDF rats, hoping to support a theoretical basis for the intestinal microbiota and lipid metabolism disorders and other related metabolic diseases.

## Materials and methods

### Ethics statement

All animal protocols and procedures were approved by the Medical Ethics Committee of Hubei Provincial Hospital of Traditional Chinese Medicine and followed the National Guidelines for laboratory Animals. All experimental procedures were implemented on the ethical guidelines for the study of experimental pain in conscious animals.

### Experimental animals

The 6-week-old male ZDF rats (fa/fa, 140 ± 11 g) and same-week-old male Zucker lean normoglycemic rats (ZLN, +/fa, 107 ± 10 g) were purchased from Vital River Laboratory Animal Technology (Beijing, China) [License No. SYXK (Beijing) 2017-033]. Rats were reared under specific disease-free conditions at 24 ± 2 °C with free access to food and water, relative humidity of 50 ± 5%, and light/dark cycles of 12 h/12 h.

### Experiment design

After 2 weeks of adaptive diet, diabetic rats were randomly divided into the following four groups: model group (MOD, N = 10), SalA low-dose group (SalA-L, N = 10), SalA medium-dose group (SalA-M, N = 10), and SalA high-dose group (SalA-H, N = 10). Another 10 lean male ZLN rats of the same week age were selected as the control group (NC, N = 10). Rats in all groups were given an ordinary diet (MD17121, Mediscience, China). Rats in the SalA-L, SalA-M, and SalA-H groups were orally treated with low dose (SalA 0.5 mg/kg/d), medium dose (SalA 1 mg/kg/d), and high dose (SalA 2 mg/kg/d) of SalA, respectively. The selection of SalA dose was based on the results of previous studies on type 1 and Type 2 DM [20, 63]. SalA (purity > 98%) was a freeze-dried powder provided by the Institute of Material Medical Chinese Academy of Medical Science (Beijing, China). Before administration, SalA was dissolved in distilled water and intragastrically administered to rats. Rats in the NC group and MOD group were intragastrically administered with an equal volume of distilled water. SalA was administered continuously for 8 weeks. General characteristics of the rats were observed and recorded during administration.

The body weight and food intake of rats in each group were measured weekly. After 7 weeks of continuous administration, oral glucose tolerance test (OGTT) was performed. After 8 weeks of continuous administration, an insulin tolerance test (ITT) was conducted and the area under the curve (AUC) was calculated. After the experiment, rats were anesthetized with 1% pentobarbital sodium and abdominal aorta blood samples were collected. The supernatant was collected after centrifugation at 180×*g* for 10 min at 4℃ and stored in a refrigerator at − 80 °C. Feces were quickly collected into sterile tubes and stored at − 80 °C until 16S rRNA gene sequencing was performed. Liver (liver weighing) and small intestine were collected rapidly on ice and frozen in liquid nitrogen or fixed with 4% paraformaldehyde for subsequent experiments.

### Measurement of fasting blood glucose (FBG), OGTT, ITT, and fasting insulin (FINS) levels

After fasting for 12 h at night, the blood glucose level in the tail vein was measured by a glucose meter (ARKRAY, Japan). The FINS levels in rats were determined by FINS enzyme-linked immunosorbent assay (ELISA) kits (TRX3565-A, Trxmark, Shanghai, China). Rats were fasted for 12 h and 4 h before OGTT and ITT tests, respectively. Glucose levels were measured at 0, 30, 60, and 120 min after oral intragastric administration of 50% glucose [2 g/kg, body weight (BW)] or subcutaneous injection of insulin (5 U/kg, BW). The AUC was calculated as follows: AUC = 0.5 × (Bg 0 min + Bg 30 min)/2 + 0.5 × (Bg 30 min + Bg 60 min)/2 + 1 × (Bg 60 min + Bg 120 min)/2. Bg represents the blood glucose level at each time point. Homeostasis model assessment of insulin resistance (HOMA-IR) index was calculated as follows: HOMA-IR = Fasting plasma glucose (mmol/L) × Fasting serum insulin (mIU/L)/22.5 [36].

### The levels of free fatty acid (FFA), lipopolysaccharide (LPS), tumor necrosis factor (TNF)-α, interleukin-6 (IL-6), total cholesterol (TC), and triglyceride (TG) were determined

The serum FFA level was determined using an automatic biochemical analyzer (Beckman Coulter Inc., CA, USA). FFA reagent was purchased from BioMerieux (Beckman). The levels of LPS (JN5237, Jining Biological Research, Shanghai, China), TNF-α (ZK-R3528, Ziker Biotech, Shenzhen, China), IL-6 (ZK-R3141, Ziker) in the serum, and TC (ZK-R3064, Ziker) and TG (ZK-R3310, Ziker) in the liver tissue were measured using ELISA kits according to the instructions.

### Hematoxylin–eosin (HE) staining

Fresh liver tissue (the right lobe of the liver) and small intestine tissue (duodenum) of rats in each group were isolated, fixed with 4% paraformaldehyde (Solarbio, Beijing, China), and embedded in paraffin. The tissues were sliced into 4-μm-thick sections. Afterwards, slides of the liver and small intestine were stained with HE staining kits (M020, Gefan Biotech, Shanghai, China). After dehydration with gradient ethanol, the slices were cleared with xylene and sealed with neutral gum, and then observed and photographed under an Olympus light microscope (Olympus, Tokyo, Japan).

### Oil red O staining

Samples from the right lobe of the liver were immobilized in 10% paraformaldehyde (Solarbio). Then, samples were embedded in Optimal Cutting Temperature (OCT) and stored at − 80 °C. Afterwards, the slices (7-μm-thick) were stained using oil red O stain solution (M067, Gefan Biotech) as per the manufacturer's instructions for the determination of fat content. Olympus BX51 optical microscope (Olympus Corporation) equipped with Olympus UPlan Apo 20× objective lens was used to observe and photograph tissue sections. Lipid contents of oil-red O-stained slices were analyzed using the Columbus Image Data Storage and Analysis System (PerkinElmer, MA, USA) and expressed as % of the lipid-positive staining areas in the area of liver tissue.

### DNA extraction and PCR amplification

After 8 weeks of continuous administration, fresh feces samples from rats in each group were collected into sterile tubes and immediately stored at − 80 °C. Total microbial DNA was extracted from feces samples using EZNA soil DNA kits (Omega Bio-Tek, GA, USA). DNA purification and concentration were determined by NanoDrop 2000 ultraviolet–visible spectrophotometer (Thermo Fisher, MA, USA) and DNA quality was evaluated by 1% (w/v) agarose gel electrophoresis. Total DNA was acted as a template for PCR amplification (95 °C for 3 min, followed by 27 cycles of 30 s at 95 °C, 30 s at 55 °C, and 45 s at 72 °C, and a final extension at 72 °C for 10 min). Specific bacterial primers 338F (5′-ACTCCTACGGGAGGCAGCAG-3′) and 806R (5′-GGACTACHVGGGTWTCTAAT-3′) were used to amplify the V3–V4 region of 16S rRNA by thermocycler PCR system (GeneAmp 9700, ABI, CA, USA). PCR products were extracted from 2% (w/v) agarose gel, further purified using the AxyPrep DNA gel extraction kits (Axygen Biosciences, CA, USA), and quantified using QuantiFluor-ST (Promega, WI, USA) according to the manufacturer’s protocol.

### Illumina Miseq sequencing and processing of sequencing

Based on the Illumina MiSeq platform (Illumina, San Diego, USA), the amplified products purified from PCR were assembled in equimolar and paired-end sequence (2 × 300). The procedure followed the standard protocols of Sasiwei Biotech (Hunan, China).

Raw fastq files were demultiplexed and low-quality sequences were filtered by Trimmomatic. They were merged using Fast Length Adjustment of Short reads in line with the following criteria: (1) when receiving an average quality score < 20 over a 50 bp sliding window, readings were truncated at any location; (2) the primers were perfectly matched (allowing two nucleotides to be mismatched), and reads containing ambiguous bases were deleted; (3) sequences with overlapping length greater than 10 bp were merged with the overlap sequence. Operational taxonomic units (OTU) were clustered with 97% sequence similarity cut-off utilizing UPARSE (V.7.1; http://drive5.com/uparse/). Chimeric sequences were identified and removed through UCHIME. The taxonomy of each sequence was evaluated according to the RDP Classifier algorithm (http://rdp.cme.msu.edu/) against the Silva (SSU123) 16S rRNA database with a confidence threshold of 70%.

### Alpha diversity index calculation

To compare the diversity of different samples, all samples in the OTU abundance matrix were randomly re-sampled (that is, “> sequence leveling processing”) according to the lowest sequencing depth, to correct the diversity differences caused by sequencing depth. Subsequently, QIIME software was employed to calculate richness estimators (Chao1), abundance-based richness estimation (ACE), and Shannon indices for each sample. The R software was applied to draw the grouped boxplot of the alpha diversity index table.

### Principal component analysis (PCA)

PCA, that is, through linear transformation, the original high-dimensional data (OTU abundance matrix of bacteria) were projected into the spatial coordinate system with lower dimensions (principal component), to achieve the purpose of dimensionality reduction and simplification of data structure, and to display the natural distribution of samples [42]. PCA analysis can extract the most important differences between samples from the original data, and order the samples in the new low-dimensional coordinate system according to these differences so that the distance of samples in the new coordinate system can restore the actual differences between samples to the greatest extent. In this sorting process, the proportion of explanations for sample differences in the original data for each axis decreases in turn. Therefore, the main distribution characteristics of community samples can be known by mapping the first two-dimensional or three-dimensional data obtained from PCA analysis, to quantify the differences and similarities between samples. PCA analysis is based on Euclidean distance to evaluate the similarity between samples, without considering the possible relationship between the original variables. R software was used for PCA analysis of community composition at the genus level, and 2D and 3D images were performed to describe the natural distribution characteristics among samples.

### Immunohistochemical staining

As described previously [30], the paraffin sections of the small intestine (5 μm) were collected for immunohistochemical staining. The sections were dewaxed with xylene, rehydrated with gradient ethanol, and then incubated overnight with Occludin primary antibody (ab216327, Abcam, Cambridge, UK) at 4 °C. Subsequently, the sections were incubated with horseradish peroxidase-labeled anti-rabbit IgG at room temperature for 30 min. Later, 3,3′-diaminobenzidine (DAB) staining, hematoxylin re-staining, and gradient ethanol dehydration were executed, and the images were observed under an Olympus microscope. Staining intensity and proportion of positive cells were used as evaluation indexes of immunostaining.

### Reverse transcription quantitative polymerase chain reaction (RT-qPCR)

Total RNA was extracted from intestinal tissue using the TRIzol reagent (Life Technologies Corporation, CA, USA). The extracted RNA was reversely transcribed into cDNA according to the instructions of the reverse transcriptase kit (ABI). Real-time quantitative PCR was carried out using TaqMan®Universal Master Mix II (ABI). PCR was carried out for 40 cycles with the following parameters: 2 min at 50 °C, 10 min at 95 °C, and for each cycle 15 s at 95 °C for denaturation and 1 min at 60 °C for annealing. Real-time qPCR assay was performed using LightCycler 480 Real-Time PCR System (Roche Applied Science, IN, USA). Glyceraldehyde-3-phosphate dehydrogenase (GAPDH) was used as an internal reference. The relative expression was calculated by the 2^−ΔΔct^ method. Primer sequences are shown in Table 1.

**Table 1:** Real-time PCR primer sequences

Gene	Forward 5′–3′	Reverse 5′–3′
Occludin	TTTGCCATAGCAGCTTTCCT	CCTGTGCAGGTTTGGTTTTT
ZO-1	GTCGCAATGGTTAACGGAGT	AGGGAGAGGAGGGAATCAAA
Claudin-1	CTGATGACCCAAGGTGGAGT	GCCACCAGGTGCAACTTAAT
GAPDH	CCGTTGTCCCAATCTGTTCT	TGTGAGGGAGATGCTCAGTG

### Statistical analysis

Statistical software SPSS 21.0 (IBM Corp. Armonk, NY, USA) and GraphPad Prism 8.01 (GraphPad Software Inc., CA, USA) were used for statistical analyses and plotting. Data were expressed as mean ± standard deviation. One-way analysis of variance (ANOVA) test was used for data comparison between groups, and Tukey's test was used for the post hoc test. Differences were considered statistically significant at *P* < 0.05.

## Results

### Effect of SalA on IR in ZDF rats

**Fig. 1:**
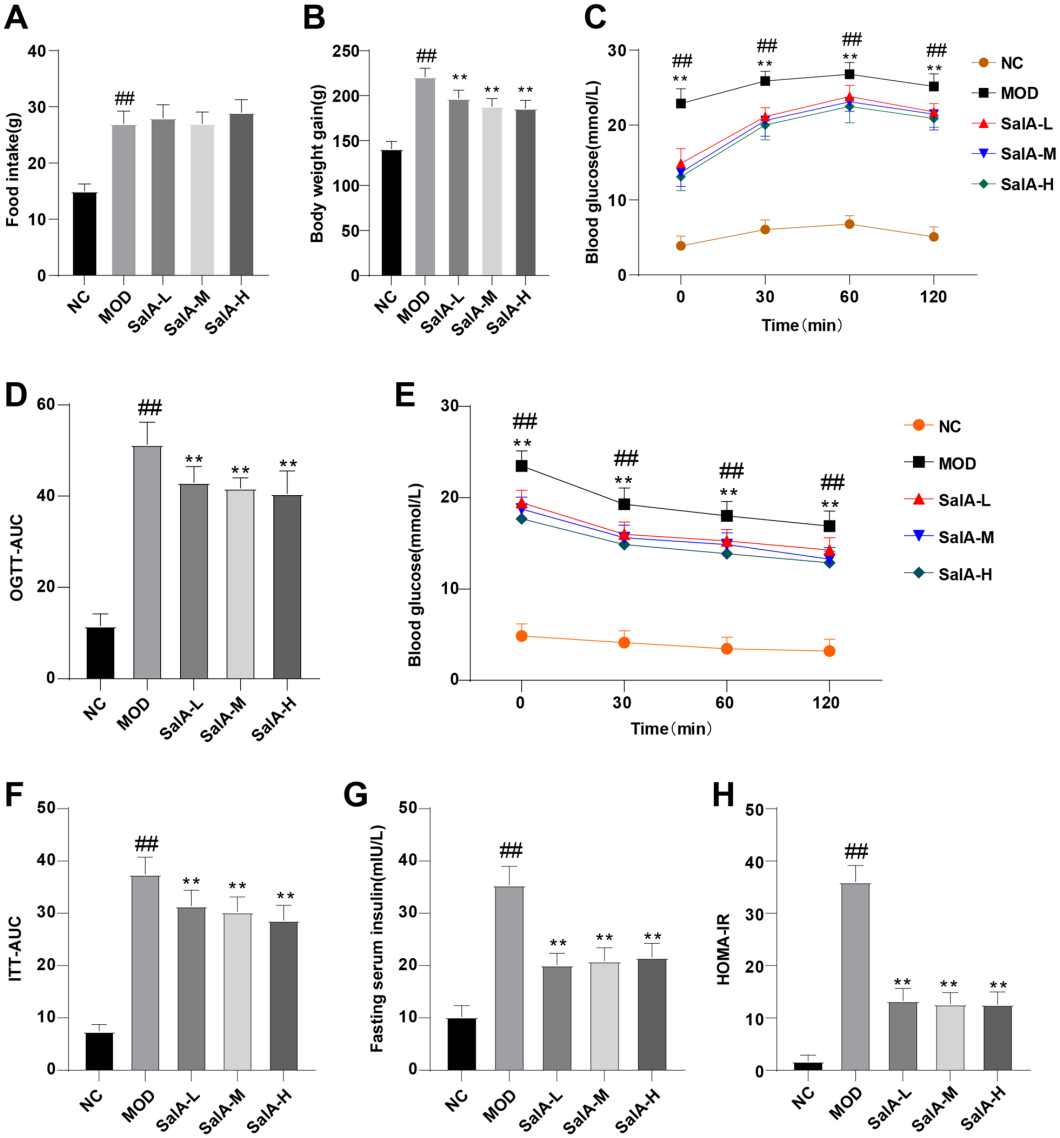
Effect of SalA on insulin resistance in ZDF rats. **A** Changes in food intake of rats in each group; **B** BW changes of rats in each group; **C** blood glucose levels during OGTT; **D** AUC of OGTT; **E** blood glucose levels during ITT; **F** AUC of ITT; **G** fasting serum insulin level; **H** HOMA-IR index. Data were expressed as mean ± standard deviation. N = 10. One-way ANOVA test was used for data comparison between two groups, and Tukey’s test was used for the post hoc test. Data comparison between the MOD group and the NC group, ^##^*P* < 0.01; data comparison between the SalA-L, SalA-M, and SalA-H groups and the MOD group, **P < 0.01

To observe the effect of SalA on ZDF rats, we recorded the changes in BW and food intake of rats in each group after 8 weeks of administration. The results revealed that after 8 weeks of administration, the food intake and BW of rats in the NC group were visibly lower than that of the MOD group (*P* < 0.01), and the food intake of rats in the SalA-L, SalA-M, and SalA-H groups was not significantly different from that of rats in the MOD group, but the BW of rats in the SalA-L, SalA-M, and SalA-H groups was less than that of the MOD group (*P* < 0.01) (Fig. 1A, B).

After 8 weeks of continuous administration, OGTT and ITT were performed. OGTT results demonstrated that blood glucose values in all groups peaked 60 min after glucose administration and then began to decline. Compared with the NC group, blood glucose and OGTT-AUC in the MOD group were elevated (*P* < 0.01); relative to the MOD group, blood glucose and OGTT-AUC in the SalA-L, SalA-M, and SalA-H groups were significantly decreased during OGTT (*P* < 0.01) (Fig. 1C, D). ITT results disclosed that blood glucose was decreased in all groups after insulin injection. Blood glucose in the SalA-L, SalA-M, and SalA-H groups was reduced during ITT compared with that in the MOD group (*P* < 0.01) (Fig. 1E). In addition, ITT-AUC in the MOD group was higher than that in the NC group (*P* < 0.01); compared with the MOD group, ITT-AUC in the SalA-L, SalA-M, and SalA-H groups was lowered (*P* < 0.01) (Fig. 1F). These results implied that SalA can improve the insulin sensitivity of ZDF rats. In addition, fasting serum insulin level and HOMA-IR index in the MOD group were higher than those in the NC group (*P* < 0.01), while SalA treatment reduced IR in ZDF rats (*P* < 0.01) (Fig. 1G, H).

### SalA can alleviate hepatic lipid accumulation and liver injury in ZDF rats

**Fig. 2:**
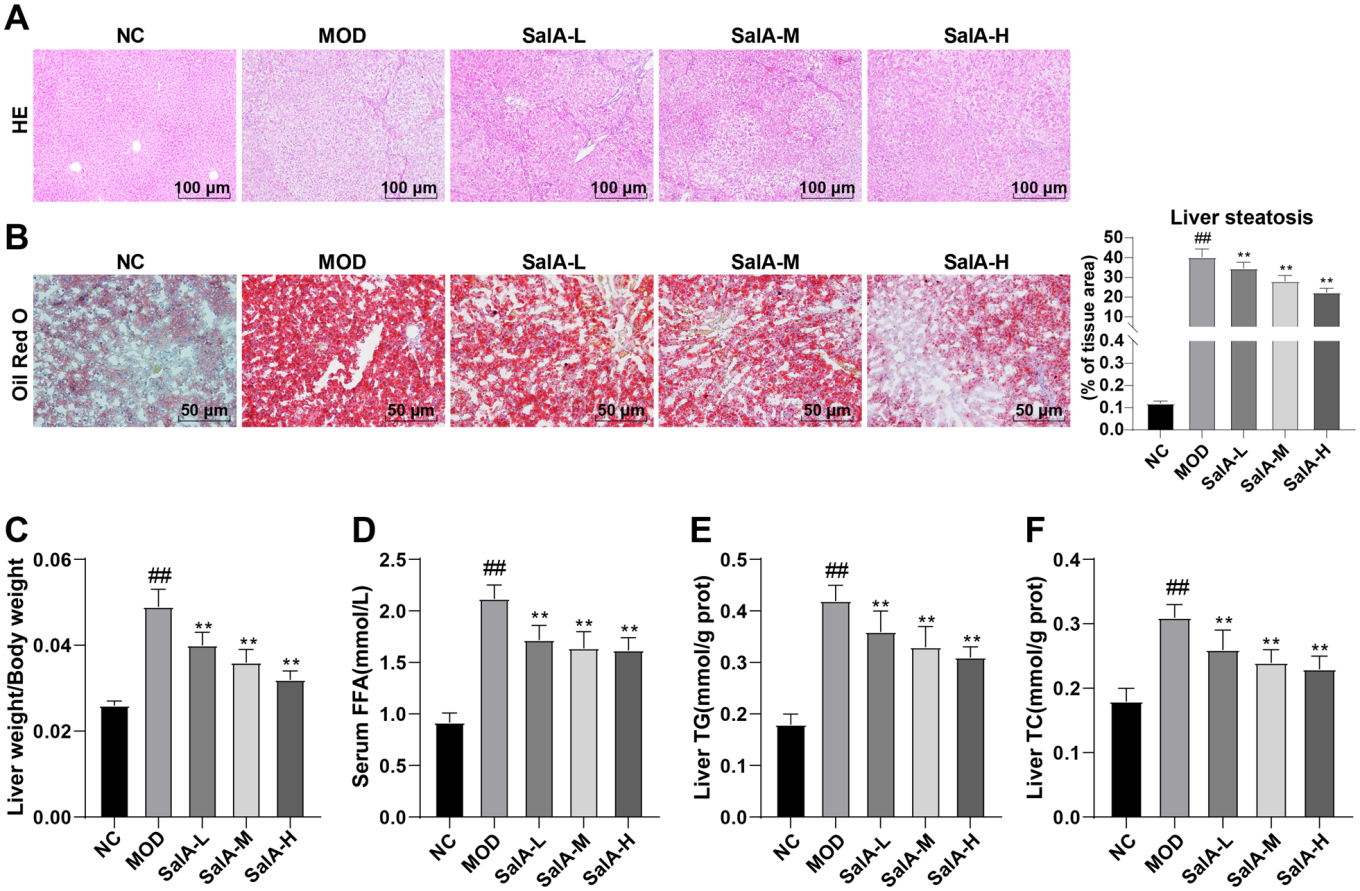
Effects of SalA on hepatic lipid metabolism and liver injury in ZDF rats. **A** HE staining of liver tissue; **B** Oil red O staining of liver tissue; **C** liver weight/BW; **D** serum FFA level; **E**, **F** liver TC and TG levels. N = 10. Data were expressed as mean ± standard deviation. One-way ANOVA test was used for data comparison between two groups, and Tukey’s test was used for the post hoc test. Data comparison between the MOD group and the NC group, ^##^*P* < 0.01; data comparison between the SalA-L, SalA-M, and SalA-H groups and the MOD group, **P < 0.01

Next, HE and oil-red O staining showed that the hepatocytes of rats in the NC group were regularly arranged radially, with normal morphology and neat arrangement, without obvious formation of large lipid droplets and inflammatory cell infiltration; hepatocytes in the MOD group showed steatosis and fat vacuoles, and their arrangement was disordered. However, after SalA treatment, hepatocytes tended to be arranged neatly without obvious fat vacuoles, indicating that SalA had a significant effect on improving liver injury in ZDF rats (Fig. 2A, B). Furthermore, the liver weight index of rats in the MOD group was higher than that of the NC group (*P* < 0.01), while the liver mass index decreased after SalA treatment (*P* < 0.01) (Fig. 2C), suggesting that SalA can visibly bring down the liver weight index. The automatic biochemical analyzer and ELISA kits uncovered that relative to the NC group, serum FFA, liver TC, and TG levels were elevated in the MOD group, which was reversed after SalA treatment (*P* < 0.01) (Fig. 2D–F).

### Influence of SalA on the overall structural changes of intestinal microbiota in ZDF rats

**Fig. 3:**
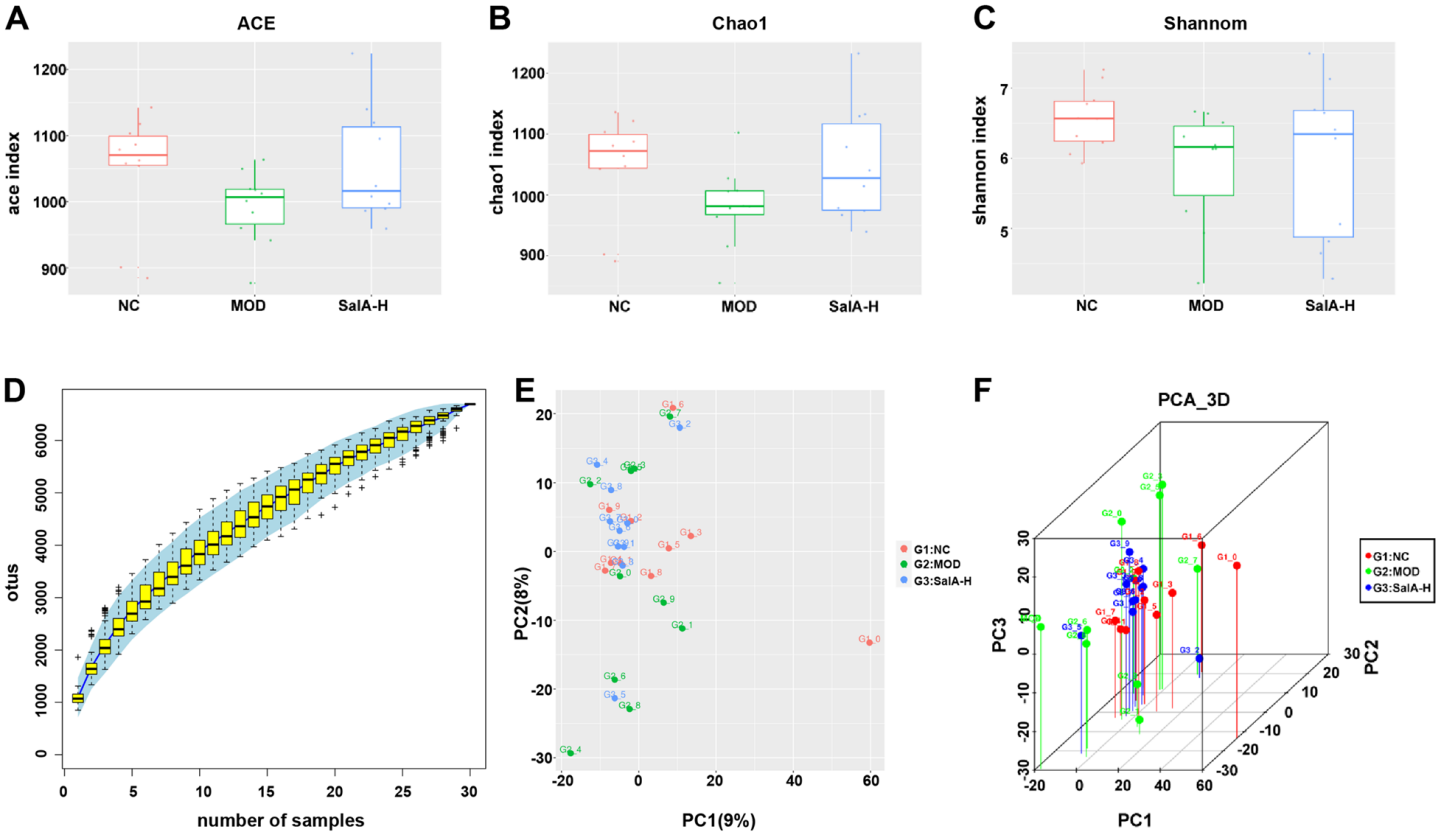
Influence of SalA-H on the overall structural changes of intestinal microbiota in ZDF rats. **A** ACE index; **B** Chao1 index; **C** Shannon index; **D** cumulative species curve; the abscissa represents the sample size, the ordinate represents the number of OTU detected, and the blue shadow reflects the confidence interval of the curve; the results reflected the increase rate of new species observed when the sample size was continuously expanded in the process of overall sampling; **E** the two-dimensional ordination diagram of community composition structure of samples analyzed by PCA. Each point represents a sample, and points of different colors belong to different groups; the closer the distance between two points, the higher the similarity of microbial community structure between two samples, the smaller the difference; the percentages in brackets on the axes represent the proportion of variation in the raw data that can be explained by the corresponding principal component. **F** The three-dimensional ordination diagram of samples analyzed by PCA. Each point represents a sample, and points of different colors belong to different groups; the closer the distance between two points is, the higher the similarity of microbial community structure between two samples is, and the smaller the difference is. N = 10

According to the original data obtained by Illumina Miseq sequencing, a total of 1,875,486 high-quality sequences were obtained from 30 samples, with an average of 62,516 sequences per sample. There were a total of 5528 OTUs, with an average of 1843 OTUs per group. There were 1887 OTUs in the NC group, 1829 OTUs in the MOD group, and 1812 OTUs in the SalA-H group (Table 2). Based on OTU results, the Alpha diversity index of each group at OTU similarity level (97%) was calculated, including ACE index and Chao1 index representing abundance assessment, as well as Shannon index representing diversity assessment. The specific results can be seen in Table 2. The larger ACE or Chao1 index is, the higher the richness of the microbiota is. The larger Shannon is, the higher the diversity of the microbiota is. Figure 3A, B displayed that the ACE index and Chao1 index of the MOD group were lower than the NC group, indicating that the bacterial abundance of ZDF rats was less than normal rats. Moreover, compared with the NC group, the Shannon value of the MOD group was declined (Fig. 3C), indicating that the bacterial diversity of ZDF rats was lower than that of the normal rats. After SalA-H administration, the bacterial richness and diversity of ZDF rats were up-regulated. Subsequently, we plotted a Specaccum species accumulation curve for the total OTU number corresponding to each sample in the OTU abundance matrix, which was adopted to measure and predict the ascent of species richness in the community with the elevation of sample size. As shown in Fig. 3D, the curve tended to be gentle, indicating that the sequencing quantity was reasonable and the majority of microorganisms can be detected. The sequencing depth can be covered to all species in the sample, which can reflect the basic situation of the number of microorganisms in the three groups. Besides, we used the PCA method to further verify the differences and changes in intestinal microbial community structure in ZDF rats under SalA-H intervention. As shown in Fig. 3E, F, the intestinal microbiota structure of rats in the NC group had the same trend, while that in the MOD group was far different. After SalA-H treatment, the intestinal microbiota structure of the SalA-H group was close to that of the NC group, indicating that the intestinal microbiota structure of the SalA-H rats recovered to a normal state.

**Table 2:** Analysis of intestinal flora abundance and diversity at 97% similarity level by high-throughput sequencing

Group	Seq number	OTU number	ACE	Chao1	Shannon
NC	31,862	1887	1048.93	1047.46	6.57
MOD	30,776	1829	992.71	981.61	5.9
SalA-H	32,662	1812	1054.08	1048.57	6.14

### Impacts of SalA on intestinal core microbial community characteristics in ZDF rats

**Fig. 4:**
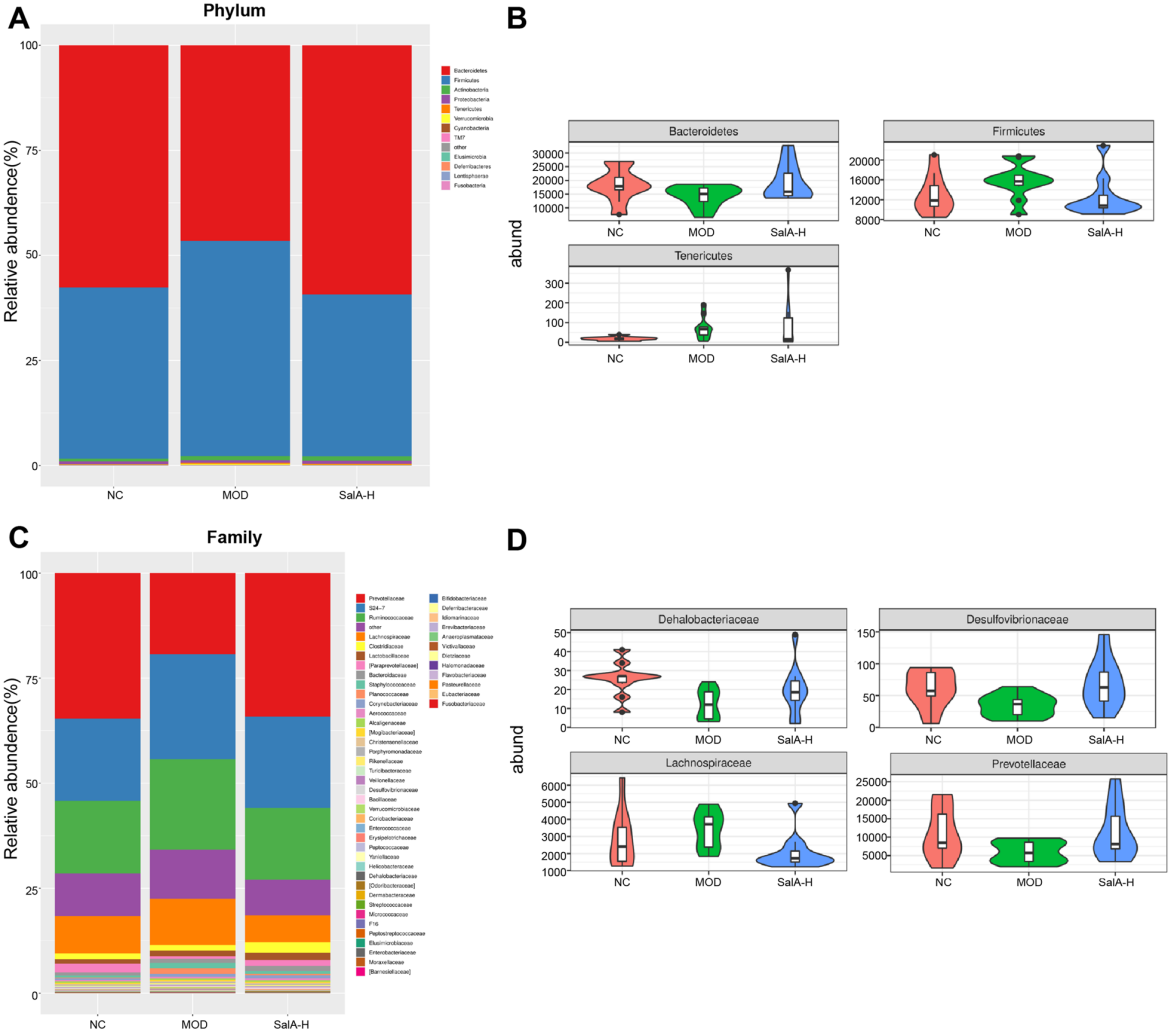
Effects of SalA on intestinal core microbial community characteristics of ZDF rats. **A** Intestinal microphyla classification in rats at the phylum level. A bar chart of the composition proportion of different groups of bacteria was drawn. The abscissa represents different groups. The ordinate represents the richness of bacteria, and different colors represent different types of bacteria. **B** Relative abundance of Bacteroidetes, Firmicutes, and Tenericutes; **C** intestinal microphyla classification in rats at the family level. A bar chart of the composition proportion of different groups of bacteria was drawn. The abscissa represents different groups, the ordinate represents the richness of bacteria, and different colors represent different types of bacteria. **D** The relative abundance of Dehalobacteriaceae, Desulfovibrionaceae, Lachnospiraceae, and Prevotellaceae. N = 10

Afterward, we investigated the bacterial composition of different groups at the taxonomic level. The results of phylum and family level were adopted to analyze the composition of intestinal microbiota in different groups of rats. At the phylum level, as shown in Fig. 4A, B, the top five bacteria in the three groups were Bacteroidetes, Firmicutes, Actinobacteria, Proteobacteria, and Tenericutes. Among them, Bacteroidetes and Firmicutes were the dominant microbiota, which accounted for the largest proportion of intestinal microbiota. Compared with the NC group, the number of Firmicutes, Actinobacteria, Tenericutes, and Verrucomicrobia was raised in the MOD group, while Bacteroidetes and Proteobacteria were lowered. After SalA-H treatment, the number of Firmicutes, Tenericutes, and Verrucomicrobia in the intestinal tract of ZDF rats was reduced, while Bacteroidetes and Proteobacteria increased, and the proportion of bacterial community was nearer to normal rats.

At the family level, the results are shown in Fig. 4C, D, and the top 4 bacteria in the 3 groups are Prevotellaceae, S24-7, Ruminococcaceae, and Lachnospiraceae. Relative to the NC group, the number of the S24-7, Ruminococcaceae, Lachnospiraceae, Staphylococcaceae, and Planococcaceae in the MOD group increased, while Prevotellaceae, Paraprevotellaceae, Dehalobacteriaceae, and Desulfovibrionaceae were down-regulated. SalA-H treatment restored intestinal microbiota in ZDF rats to a certain extent, and the proportion of intestinal microbiota in the SALA-H group was closer to that in the NC group.

### SalA can reduce intestinal epithelial barrier injury and LPS and inflammatory cytokines levels in ZDF rats

**Fig. 5:**
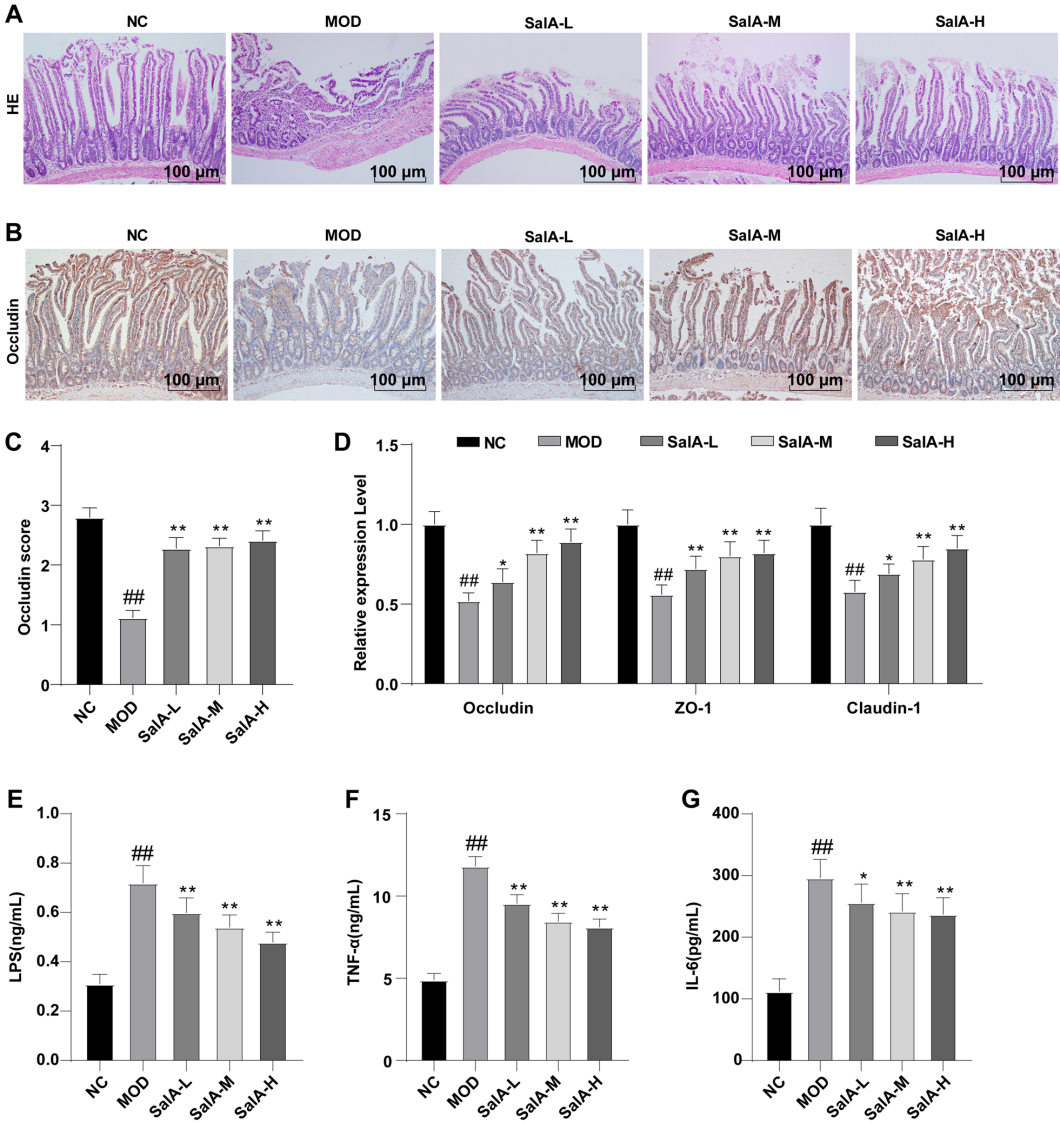
Effects of SalA on intestinal tissue, LPS, and inflammatory factors. **A**, **B** HE staining and immunohistochemical staining of intestinal tissue samples; **C** Occludin expression score; **D** RT-qPCR was used to detect the expression of tight junction proteins ZO-1, Occludin, and Claudin-1 in intestinal tissue, as well as the levels of **E** LPS, **F** TNF-α and **G** IL-6 in the serum. Data were expressed as mean ± standard deviation. N = 10. One-way ANOVA test was used for data comparison between two groups, and Tukey’s test was used for the post hoc test. Data comparison between the MOD group and the NC group, ^##^*P* < 0.01; data comparison between the SalA-L, SalA-M, and SalA-H groups and the MOD group, *P < 0.05, **P < 0.01

Moreover, we detected the effect of SalA on the small intestine by HE staining and found that the small intestine structure of rats in the NC group was intact, and the villi of the small intestine were arranged neatly. Compared with the NC group, intestinal villi in the MOD group were disordered, collapsed, and recessed. After SalA treatment, the small intestine morphology of the MOD group was overtly improved (Fig. 5A). Apparently, SalA can alleviate intestinal epithelial villus injury in ZDF rats. Subsequently, to further ascertain the effect of SalA on the permeability of intestinal epithelium in ZDF rats, immunohistochemical staining was performed on the small intestine of rats to detect the expression of tight junction protein Occludin. The small intestine of rats in the NC group was deeply stained and Occludin protein level was enhanced. Compared with the NC group, the staining color in the MOD group became shallow and the protein expression of Occludin decreased. After SalA treatment, the expression of Occludin increased compared with the MOD group (*P* < 0.01) (Fig. 5B, C). In addition, RT-qPCR showed that the expression of tight junction proteins ZO-1, Occludin, and Claudin-1 in the MOD group was lower than that in the NC group. SalA increased the expression of tight junction protein (*P* < 0.05) (Fig. 5D). Besides, the comparison of serum LPS, IL-6, and TNF-α levels revealed that the levels of LPS, IL-6, and TNF-α in the MOD group were higher than those in the NC group, while the levels of LPS, IL-6, and TNF-α were reduced in a dose-dependent manner after SalA treatment (*P* < 0.05) (Fig. 5E–G).

## Discussion

DM remains a global public health threat and the incidence continues to rise with the progressive elevation in obesity and the aging population [39]. The contributors of IR to T2DM include impaired insulin signaling and complicated interplay of multifaceted metabolic pathways, and more importantly, the metabolites and microbiota can regulate insulin sensitivity [59]. SalA is a neuroprotective extract of *Salvia miltiorrhiza* with the functions of anti-apoptosis and anti-inflammation [61], and *Salvia miltiorrhiza* is potent medicine in DM treatment [18, 22]. This study highlighted the regulation of SalA on intestinal microbiota and lipid metabolism in ZDF rats.

IR is considered a pivotal cause of T2DM onset [44] and declined insulin clearance is linked with IR-related T2DM [15]. Moreover, obesity is a risk factor for T2DM [35]. To observe the effect of SalA on ZDF rats, SalA was administered to ZDF rats for 8 weeks. Afterward, we found that different doses of SalA inhibited the elevation of weight, fasting serum insulin, and HOMA-IR in ZDF rats. OGTT and ITT assays showed that SalA treatment diminished blood glucose, OGTT-AUC, and ITT-AUC in ZDF rats. Similarly, SalB, another potent active water-soluble component in *Salvia miltiorrhiza*, impedes PPARγ expression and adipogenesis, thus attenuating weight gain and obesity-associated metabolic disorders [51]. SalB improves IR of ob/ob mice by suppressing endoplasmic reticulum stress in the liver [45]. SalA exhibits the antidiabetic properties in diabetic animal models by improving mitochondrial function, raising ATP production, and lowering MMP via the CaMKKβ/AMPK pathway, embodied by diminishing FBG and improving glucose tolerance [40]. Additionally, a study has highlighted that α-glucosidase is an important drug target in the treatment of type 2 diabetes, and inhibition of α-glucosidase can reduce the absorption of glucose, whereas SalA inhibits α-glucosidase activity [47, 48]. Accordingly, SalA may reduce glucose concentration by inhibiting the activity of α-glucosidase. As a whole, the aforementioned finding highlighted that SalA can alleviate hyperinsulinemia in ZDF rats.

Adipose tissue is construed as an endocrine organ that is closely related to the body’s energy production, consumption, and metabolism [24]. Augmented visceral fat lipolysis through adipose tissue lipoprotein lipase stimulates the excessive generation of FFA, resulting in IR and metabolic diseases, such as T2DM [11, 23]. Insulin signaling is essential for maintaining adipose tissue function [9]. During insulin-resistant status such as T2DM, insulin fails to inhibit hepatic glucose generation but potentiates lipid synthesis resulting in hypertriglyceridemia and hyperglycemia [49]. Hyperlipidemia is characterized by elevated TC and TG [31]. Additionally, DM facilitates liver injury by triggering inflammation and fibrosis via increasing mitochondrial oxidative stress [43], and the liver is imperative in modulating insulin clearance and glucose metabolism [16]. Therefore, we analyzed the relevant parameters of the liver and then observed that SalA treatment palliated the liver injury in ZDF rats. In addition, our results indicated that SalA decreased the liver weight index, serum FFA, liver TC, and liver TG levels in ZDF rats. Similarly, SalA can decrease hepatic TG, alleviate hepatic fibrosis, and ameliorate hepatic mitochondrial function in diabetic animals [40, 41]. SalA treatment robustly mitigates obesity and liver damage in rats and reduces lipid accumulation in the liver in high-fat diet-fed rats [13]. Moreover, SalB reduces TG, FFA, and serum insulin levels, down-regulates hepatic gluconeogenic gene, and improves insulin intolerance, thus ameliorating dyslipidemia and hyperglycemia in db/db mice via the AMPK pathway [21]. Collectively, SalA ameliorated hepatic lipid accumulation and liver injury in ZDF rats.

T2DM remains an acknowledged social and public health issue that may be caused by intestinal microbiota imbalance through inflammatory responses [32, 34]. T2DM rats present imbalanced intestinal microbiota, obvious pathological alternation of the intestinal mucosa, aggravated inflammatory responses, and weakened antioxidant ability [27]. Intriguingly, salvianolic acids probably exert medical effects by regulating intestinal bacteria [5]. Afterward, we estimated the effect of SalA on the structure of intestinal microbiota in ZDF rats and found that high-dose of SalA increased the microbiota richness and diversity and normalized the intestinal microbiota structure in ZDF rats. Moreover, the analyses at the phylum and family levels unraveled that high-dose of SalA restored intestinal microbiota and made the proportion of intestinal microbiota in ZDF rats closer to that in normal rats. Consistently, SalA can regulate the gut microbiota imbalance during colitis by augmenting the gut microbial diversity and selectively promoting several probiotic populations [50]. Furthermore, SalB regulates intestinal microbiota composition by inhibiting the segregation in gut microbial clusters of obese mice from normal mice and shifting these clusters close to those in normal mice [28]. Altogether, SalA could improve the structure of intestinal microbiota and maintain the homeostasis of intestinal core microbiota in ZDF rats.

DM is implicated with a dysfunctional intestinal barrier and a raised risk for inflammation and systemic infection in people [8]. Increased permeability of the intestinal epithelial barrier is linked to metabolic homeostasis disruption causing obesity and T2DM [37]. Through alterations in intestinal permeability, the intestinal barrier is impaired whereby the access of dietary antigens and infectious agents to mucosal immune elements is promoted, resulting in immune reactions, elevated cytokine generation, and consequent IR [10]. Moreover, intracellular serine/threonine kinases activated by inflammation factors can catalyze the inhibitory phosphorylation of essential proteins of the insulin signaling, resulting in IR [7]. Next, we investigated the association between SalA and the intestinal barrier. HE results indicated that SalA extenuated intestinal epithelial villous injury in ZDF rats. Afterward, our results showed that SalA could increase ZO-1, Occludin, and Claudin-1 expressions and decrease LPS, IL-6, and TNF-α levels. Accordingly, the preceding report has noted that DM diminishes tight junction proteins, including ZO-1, occludin, and claudin-1, and increases intestinal permeability to damage the intestinal barrier [60]. Additionally, the decline in intestinal permeability is positively-associated with LPS levels and DM patients exhibit elevated plasma LPS [17, 46]. As reported, SalA confers potent protection against endothelial injury and it can attenuate ischemia/reperfusion-triggered endothelial hyperpermeability and endothelial barrier dysfunction by suppressing VLDL receptor expression [57]. SalA can protect against early-stage diabetic nephropathy by improving glomerular endothelial hyperpermeability in T2DM rats, which can also alleviate inflammation and restore the disturbed autophagy in diabetic rats and glomerular endothelial cells through the AGE-RAGE-Nox4 axis [20]. More importantly, SalA reduces serum hs-CRP levels and inhibits the activation of the NF-κB pathway and NLRP3 inflammasome in aortic tissues of ZDF rats, indicating its anti-inflammatory role [33]. Conjointly, SalA decreased LPS and inflammatory cytokine levels in ZDF rats, thus alleviating the damage to the intestinal epithelial barrier.

In summary, our findings elucidated that SalA can relieve hyperinsulinemia, reduce liver injury, improve lipid metabolism, maintain intestinal microbiota balance and reduce intestinal epithelial barrier injury in ZDF rats. However, this is merely a rudimentary study and the results lack further clinical data. Future studies should explore the specific mechanism of SalA in modulating intestinal microbiota and lipid metabolism in ZDF rats and the regulation of other Chinese herbal medicines. Furthermore, conducting clinical studies is also worthwhile.

## Data Availability

All the data generated or analyzed during this study are included in this published article.

## Abbreviations

DM: Diabetes mellitus
T2DM: Type 2 diabetes mellitus
IR: Insulin resistance
ZDF: Zucker diabetic fatty
FFA: Free fatty acid
SalA: Salvianolic acid A
ZLN: Zucker lean normoglycaemic
OGTT: Oral glucose tolerance test
ITT: Insulin tolerance test
FINS: Fasting insulin
TC: Total cholesterol
TG: Triglyceride
FFA: Free fatty acid
FBG: Fasting blood glucose
BW: Body weight
LPS: Lipopolysaccharide
TNF-α: Tumor necrosis factor
IL-6: Interleukin-6
HE: Hematoxylin–eosin
OUT: Operational taxonomic units
Chao1: Richness estimators
ACE: Abundance-based richness estimation
PCA: Principal component analysis
DAB: 3,3′-Diaminobenzidine
RT-qPCR: Reverse transcription quantitative polymerase chain reaction

## References

[1] Agrawal A, Narayan G, Gogoi R, Thummer RP (2021). Recent advances in the generation of beta-cells from induced pluripotent stem cells as a potential cure for diabetes mellitus. Adv Exp Med Biol.

[2] Ahmed SAH, Ansari SA, Mensah-Brown EPK, Emerald BS (2020). The role of DNA methylation in the pathogenesis of type 2 diabetes mellitus. Clin Epigenet.

[3] Almugadam BS, Yang P, Tang L (2021). Analysis of jejunum microbiota of HFD/STZ diabetic rats. Biomed Pharmacother.

[4] Balakumar M, Prabhu D, Sathishkumar C, Prabu P, Rokana N, Kumar R (2018). Improvement in glucose tolerance and insulin sensitivity by probiotic strains of Indian gut origin in high-fat diet-fed C57BL/6J mice. Eur J Nutr.

[5] Cai H, Su S, Li Y, Zhu Z, Guo J, Zhu Y (2019). Danshen can interact with intestinal bacteria from normal and chronic renal failure rats. Biomed Pharmacother.

[6] Cho NH, Shaw JE, Karuranga S, Huang Y, da Rocha Fernandes JD, Ohlrogge AW (2018). IDF diabetes atlas: global estimates of diabetes prevalence for 2017 and projections for 2045. Diabetes Res Clin Pract.

[7] Coope A, Torsoni AS, Velloso LA (2016). Mechanisms in endocrinology: metabolic and inflammatory pathways on the pathogenesis of type 2 diabetes. Eur J Endocrinol.

[8] Crakes KR, Pires J, Quach N, Ellis-Reis RE, Greathouse R, Chittum KA (2021). Fenofibrate promotes PPARalpha-targeted recovery of the intestinal epithelial barrier at the host-microbe interface in dogs with diabetes mellitus. Sci Rep.

[9] Czech MP (2017). Insulin action and resistance in obesity and type 2 diabetes. Nature Medicine.

[10] de Kort S, Keszthelyi D, Masclee AA (2011). Leaky gut and diabetes mellitus: what is the link?. Obes Rev.

[11] Den Hartogh DJ, Vlavcheski F, Giacca A, MacPherson RE, Tsiani E (2022). Carnosic acid attenuates the free fatty acid-induced insulin resistance in muscle cells and adipocytes. Cells.

[12] Dilworth L, Facey A, Omoruyi F (2021). Diabetes mellitus and its metabolic complications: the role of adipose tissues. Int J Mol Sci.

[13] Ding C, Zhao Y, Shi X, Zhang N, Zu G, Li Z (2016). New insights into salvianolic acid A action: regulation of the TXNIP/NLRP3 and TXNIP/ChREBP pathways ameliorates HFD-induced NAFLD in rats. Sci Rep.

[14] Feng S, Cong H, Ji L (2020). Salvianolic acid A exhibits anti-inflammatory and antiarthritic effects via inhibiting NF-kappaB and p38/MAPK pathways. Drug Des Devel Ther.

[15] Fiorentino TV, Sesti F, Succurro E, Pedace E, Andreozzi F, Sciacqua A (2018). Higher serum levels of uric acid are associated with a reduced insulin clearance in non-diabetic individuals. Acta Diabetol.

[16] Guerra S, Gastaldelli A (2020). The role of the liver in the modulation of glucose and insulin in non alcoholic fatty liver disease and type 2 diabetes. Curr Opin Pharmacol.

[17] Guo Y, Liu CQ, Liu GP, Huang ZP, Zou DJ (2019). Roux-en-Y gastric bypass decreases endotoxemia and inflammatory stress in association with improvements in gut permeability in obese diabetic rats. J Diabetes.

[18] Guo Y, Sun J, Zhang R, Yang P, Zhang S, Wu Z (2021). *Salvia miltiorrhiza* improves type 2 diabetes: a protocol for systematic review and meta-analysis. Medicine (Baltimore).

[19] Haro C, Montes-Borrego M, Rangel-Zuniga OA, Alcala-Diaz JF, Gomez-Delgado F, Perez-Martinez P (2016). Two healthy diets modulate gut microbial community improving insulin sensitivity in a human obese population. J Clin Endocrinol Metab.

[20] Hou B, Qiang G, Zhao Y, Yang X, Chen X, Yan Y (2017). Salvianolic acid A protects against diabetic nephropathy through ameliorating glomerular endothelial dysfunction via inhibiting AGE-RAGE signaling. Cell Physiol Biochem.

[21] Huang MQ, Zhou CJ, Zhang YP, Zhang XQ, Xu W, Lin J (2016). Salvianolic acid B ameliorates hyperglycemia and dyslipidemia in db/db mice through the AMPK pathway. Cell Physiol Biochem.

[22] Jia Q, Zhu R, Tian Y, Chen B, Li R, Li L (2019). *Salvia miltiorrhiza* in diabetes: a review of its pharmacology, phytochemistry, and safety. Phytomedicine.

[23] Ko S-H, Jung YJ (2021). Energy metabolism changes and dysregulated lipid metabolism in postmenopausal women. Nutrients.

[24] Kusminski CM, Bickel PE, Scherer PE (2016). Targeting adipose tissue in the treatment of obesity-associated diabetes. Nat Rev Drug Discov.

[25] Lee SR, Jeon H, Kwon JE, Suh H, Kim BH, Yun MK (2020). Anti-osteoporotic effects of *Salvia miltiorrhiza* Bunge EtOH extract both in ovariectomized and naturally menopausal mouse models. J Ethnopharmacol.

[26] Li H, Cheng Y, Dong H, Wang X, Li J, Gao Q (2016). Preparation of salvianolic acid A by the degradation reaction of salvianolic acid B in subcritical water integrated with pH-zone-refining counter-current chromatography. J Chromatogr A.

[27] Li J, Zhang H, Wang G (2020). Correlations between inflammatory response, oxidative stress, intestinal pathological damage and intestinal flora variation in rats with type 2 diabetes mellitus. Eur Rev Med Pharmacol Sci.

[28] Li L, Li R, Zhu R, Chen B, Tian Y, Zhang H (2020). Salvianolic acid B prevents body weight gain and regulates gut microbiota and LPS/TLR4 signaling pathway in high-fat diet-induced obese mice. Food Funct.

[29] Li M, Ding L, Hu YL, Qin LL, Wu Y, Liu W (2021). Herbal formula LLKL ameliorates hyperglycaemia, modulates the gut microbiota and regulates the gut-liver axis in Zucker diabetic fatty rats. J Cell Mol Med.

[30] Li M, Chai HF, Peng F, Meng YT, Zhang LZ, Zhang L (2018). Estrogen receptor beta upregulated by lncRNA-H19 to promote cancer stem-like properties in papillary thyroid carcinoma. Cell Death Dis.

[31] Li X, Hu X, Pan T, Dong L, Ding L, Wang Z (2021). Kanglexin, a new anthraquinone compound, attenuates lipid accumulation by activating the AMPK/SREBP-2/PCSK9/LDLR signalling pathway. Biomed Pharmacother.

[32] Lv M, Li L, Li W, Yang F, Hu Q, Xiong D (2021). Mechanism research on the interaction regulation of Escherichia and IFN-gamma for the occurrence of T2DM. Ann Palliat Med.

[33] Ma Q, Yang Q, Chen J, Yu C, Zhang L, Zhou W (2020). Salvianolic acid A ameliorates early-stage atherosclerosis development by inhibiting NLRP3 inflammasome activation in zucker diabetic fatty rats. Molecules.

[34] Ma Q, Li Y, Li P, Wang M, Wang J, Tang Z (2019). Research progress in the relationship between type 2 diabetes mellitus and intestinal flora. Biomed Pharmacother.

[35] Malone JI, Hansen BC (2019). Does obesity cause type 2 diabetes mellitus (T2DM)? Or is it the opposite?. Pediatr Diabetes.

[36] Matthews DR, Hosker JP, Rudenski AS, Naylor BA, Treacher DF, Turner RC (1985). Homeostasis model assessment: insulin resistance and beta-cell function from fasting plasma glucose and insulin concentrations in man. Diabetologia.

[37] Olivier S, Pochard C, Diounou H, Castillo V, Divoux J, Alcantara J (2021). Deletion of intestinal epithelial AMP-activated protein kinase alters distal colon permeability but not glucose homeostasis. Mol Metab.

[38] Patel TP, Rawal K, Bagchi AK, Akolkar G, Bernardes N, Dias DDS (2016). Insulin resistance: an additional risk factor in the pathogenesis of cardiovascular disease in type 2 diabetes. Heart Fail Rev.

[39] Perez-Cremades D, Chen J, Assa C, Feinberg MW (2022). MicroRNA-mediated control of myocardial infarction in diabetes. Trends Cardiovasc Med.

[40] Qiang G, Yang X, Shi L, Zhang H, Chen B, Zhao Y (2015). Antidiabetic effect of salvianolic acid A on diabetic animal models via AMPK activation and mitochondrial regulation. Cell Physiol Biochem.

[41] Qiang G, Yang X, Xuan Q, Shi L, Zhang H, Chen B (2014). Salvianolic acid A prevents the pathological progression of hepatic fibrosis in high-fat diet-fed and streptozotocin-induced diabetic rats. Am J Chin Med.

[42] Ramette A (2007). Multivariate analyses in microbial ecology. FEMS Microbiol Ecol.

[43] Sharma K, Zajc I, Ziberna L (2021). Dietary vitamin D equilibrium in serum ameliorates direct bilirubin associated diabetes mellitus. Chem Biol Interact.

[44] Sharma S, Taliyan R (2016). Histone deacetylase inhibitors: future therapeutics for insulin resistance and type 2 diabetes. Pharmacol Res.

[45] Shi Y, Pan D, Yan L, Chen H, Zhang X, Yuan J (2020). Salvianolic acid B improved insulin resistance through suppression of hepatic ER stress in ob/ob mice. Biochem Biophys Res Commun.

[46] Singh A, Boden G, Rao AK (2015). Tissue factor and Toll-like receptor (TLR)4 in hyperglycaemia-hyperinsulinaemia. Effects in healthy subjects, and type 1 and type 2 diabetes mellitus. Thromb Haemost.

[47] Tang H, Zhao D, Xue Z (2018). Exploring the interaction between *Salvia miltiorrhiza* and alpha-glucosidase: insights from computational analysis and experimental studies. RSC Adv.

[48] Tang H, Ma F, Zhao D (2019). Integrated multi-spectroscopic and molecular modelling techniques to probe the interaction mechanism between salvianolic acid A and alphaglucosidase. Spectrochim Acta A Mol Biomol Spectrosc.

[49] Titchenell PM, Lazar MA, Birnbaum MJ (2017). Unraveling the regulation of hepatic metabolism by insulin. Trends Endocrinol Metab.

[50] Wang K, Yang Q, Ma Q, Wang B, Wan Z, Chen M (2018). Protective effects of salvianolic acid A against dextran sodium sulfate-induced acute colitis in rats. Nutrients.

[51] Wang P, Xu S, Li W, Wang F, Yang Z, Jiang L (2014). Salvianolic acid B inhibited PPARgamma expression and attenuated weight gain in mice with high-fat diet-induced obesity. Cell Physiol Biochem.

[52] Wei X, Zong W, Gao Y, Peng S, Liu K, Zheng Y (2020). Effects of the traditional chinese medicine tang luo ning on intestinal flora and oxidative stress in diabetic rats. Evid Based Complement Alternat Med.

[53] Xia C, Zhang X, Cao T, Wang J, Li C, Yue L (2020). Hepatic transcriptome analysis revealing the molecular pathogenesis of type 2 diabetes mellitus in Zucker diabetic fatty rats. Front Endocrinol (Lausanne).

[54] Xu C, Hou B, He P, Ma P, Yang X, Yang X (2020). Neuroprotective effect of salvianolic acid A against diabetic peripheral neuropathy through modulation of Nrf2. Oxid Med Cell Longev.

[55] Xu H, Li Y, Che X, Tian H, Fan H, Liu K (2014). Metabolism of salvianolic acid A and antioxidant activities of its methylated metabolites. Drug Metab Dispos.

[56] Xu J, Hirai T, Koya D, Kitada M (2021). Effects of SGLT2 inhibitors on atherosclerosis: lessons from cardiovascular clinical outcomes in type 2 diabetic patients and basic researches. J Clin Med.

[57] Yang D, Zhang P, Wang T, Gao L, Qiao Z, Liang Y (2014). SalA attenuates ischemia/reperfusion-induced endothelial barrier dysfunction via down-regulation of VLDL receptor expression. Cell Physiol Biochem.

[58] Yang L, Jiang L, Jiang D, Liu B, Jin S (2019). The protective effects of salvianolic acid A against hepatic ischemia-reperfusion injury via inhibiting expression of toll-like receptor 4 in rats. Arch Med Sci.

[59] Yang Q, Vijayakumar A, Kahn BB (2018). Metabolites as regulators of insulin sensitivity and metabolism. Nat Rev Mol Cell Biol.

[60] Yang Y, Zhao M, He X, Wu Q, Li DL, Zang WJ (2021). Pyridostigmine protects against diabetic cardiomyopathy by regulating vagal activity, gut microbiota, and branched-chain amino acid catabolism in diabetic mice. Front Pharmacol.

[61] Yang Y, Song J, Liu N, Wei G, Liu S, Zhang S (2022). Salvianolic acid A relieves cognitive disorder after chronic cerebral ischemia: involvement of Drd2/Cryab/NF-kappaB pathway. Pharmacol Res.

[62] Yin Z, Wang X, Yang X, Chen Y, Duan Y, Han J (2021). *Salvia miltiorrhiza* in anti-diabetic angiopathy. Curr Mol Pharmacol.

[63] Yu X, Zhang L, Yang X, Huang H, Huang Z, Shi L (2012). Salvianolic acid A protects the peripheral nerve function in diabetic rats through regulation of the AMPK-PGC1alpha-Sirt3 axis. Molecules.

[64] Yu XY, Zhang L, Yang XY, Li XT, Du GH (2016). Salvianolic acid A improves intestinal motility in diabetic rats through antioxidant capacity and upregulation of nNOS. J Dig Dis.

[65] Zhang C, Zhang D, Wang H, Lin Q, Li M, Yuan J (2022). Hyperbaric oxygen treatment improves pancreatic betacell function and hepatic gluconeogenesis in STZinduced type2 diabetes mellitus model mice. Mol Med Rep.

